# Intravenous Magnesium: Prompt Use for Asthma in Children Treated in the Emergency Department (IMPACT-ED): Protocol for a Multicenter Pilot Randomized Controlled Trial

**DOI:** 10.2196/48302

**Published:** 2023-07-17

**Authors:** Michael D Johnson, Bradley J Barney, Joseph E Rower, Yaron Finkelstein, Joseph J Zorc

**Affiliations:** 1 Division of Pediatric Emergency Medicine Department of Pediatrics University of Utah School of Medicine Salt Lake City, UT United States; 2 Division of Pediatric Critical Care Department of Pediatrics University of Utah School of Medicine Salt Lake City, UT United States; 3 Department of Pharmacology and Toxicology University of Utah College of Pharmacy Salt Lake City, UT United States; 4 Division of Emergency Medicine Hospital for Sick Children University of Toronto Toronto, ON Canada; 5 Division of Clinical Pharmacology and Toxicology Hospital for Sick Children University of Toronto Toronto, ON Canada; 6 Department of Pediatrics Children's Hospital of Philadelphia University of Pennsylvania Philadelphia, PA United States

**Keywords:** asthma, child, emergency service, feasibility studies, hospitalization, hypotension, magnesium, multicenter studies, randomized controlled trials

## Abstract

**Background:**

Children managed for asthma in an emergency department (ED) may be less likely to be hospitalized if they receive intravenous magnesium sulfate (IVMg). Asthma guidelines recommend IVMg for severely sick children but note a lack of evidence to support this recommendation. All previous trials of IVMg in children with asthma have been too small to answer whether IVMg is effective and safe. A few major questions remain about IVMg. First, it has not been tested early in the course of ED treatment, when the impact on hospitalization would be greatest. Second, the clinical impact of hypotension, a known adverse effect of IVMg, has not been well characterized in previous research. Third, no trials have compared different IVMg doses or serial serum magnesium (total and ionized) concentrations to optimize dosing, so the most effective dose is unknown. A large, conclusive, randomized, placebo-controlled clinical trial of IVMg might be challenging due to the need to enroll and complete study procedures quickly, a lack of understanding of blood pressure changes after IVMg, and a lack of pharmacologic information to guide the optimal doses of IVMg to be tested. Therefore, a pilot study to inform the above gaps is warranted before conducting a definitive trial.

**Objective:**

The objectives of this study are to (1) demonstrate the feasibility of enrolling children with severe acute asthma in the ED in a multicenter, randomized controlled trial of a placebo, low-dose IVMg, or high-dose IVMg; (2) demonstrate the feasibility of timely delivery of study medication, assessment of blood pressure, and evaluation of adverse events in a standardized protocol; and (3) externally validate a previously constructed pharmacokinetic model and develop a combined pharmacokinetic/pharmacodynamic model for IVMg using magnesium (total and ionized) serum concentrations and their correlation with measures of efficacy and safety.

**Methods:**

This pilot trial tests procedures and gathers information to plan a definitive trial. The pilot trial will enroll as many as 90 children across 3 sites, randomize each child to 1 of 3 study arms, measure blood pressure frequently, and collect 3 blood samples from each participant with corresponding clinical asthma scores.

**Results:**

The project was funded by the National Heart, Lung, and Blood Institute (1 R34HL152047-2) in March 2022. Enrollment began in September 2022, and 43 children have been enrolled as of April 2023. We will submit the results for publication in late 2023.

**Conclusions:**

The results of this study will guide the planning of a large, definitive, multicenter trial powered to evaluate if IVMg reduces hospitalization. Blood pressure measurements will inform a monitoring plan for the larger trial, and blood samples and asthma scores will be used to validate pharmacologic models to select the optimal dose of IVMg to be evaluated in the definitive trial.

**Trial Registration:**

ClinicalTrials.gov NCT05166811; https://clinicaltrials.gov/ct2/show/NCT05166811

**International Registered Report Identifier (IRRID):**

DERR1-10.2196/48302

## Introduction

### Background

Asthma is the most common chronic illness in childhood and a leading cause of pediatric hospitalization, disability, and health care–related costs [1]. The current standard management of acute asthma in children is defined in guidelines published in 2007 by the National Heart, Lung, and Blood Institute (NHLBI) and includes inhaled bronchodilators (albuterol and ipratropium), systemic corticosteroids, oxygen, and further treatment depending on response [2]. Although these guidelines have been broadly implemented, hospitalization remains a common outcome for children with asthma after emergency department (ED) treatment, ranging from 13% to 50% of all asthma visits among pediatric EDs nationally [3].

Intravenous magnesium sulfate (IVMg) has been used to treat asthma for more than 70 years [4,5] and may reduce hospitalization in children with asthma. However, its specific role in treating pediatric acute asthma is still unclear [6]. NHLBI asthma guidelines suggest that clinicians consider a 20-minute infusion of IVMg in children or adults as an adjunct for life-threatening exacerbations, or for severe distress after an hour of initial therapy. These guidelines do not suggest a specific dose but rather provide a broad 3-fold dose range of 25-75 mg/kg. Conflicting evidence prevents a definitive recommendation or direction for clinical implementation [2]. In a review of care delivered to children with asthma over 6 years in 7 EDs, 5774 (25.7%) of 22,499 children hospitalized for asthma received IVMg in the ED [7]. A meta-analysis of previous small trials suggests that IVMg given in the ED may reduce hospitalization in children by 30% [8]. Applying a 30% reduction in hospitalization to the remaining hospitalized children who do not receive IVMg in the United States could potentially avoid 16,500 hospitalizations each year, each at an average cost of US $3600 [9], producing direct cost savings of US $65 million yearly in addition to avoiding indirect costs of missed school and work.

In this project, we are conducting a pilot trial that will address the following knowledge gaps needed to conduct a conclusive trial to assess the impact of IVMg on hospitalization.

First, previous trials evaluated IVMg for acute asthma only in children refractory to initial treatment. Though IVMg is currently infrequently used in clinical practice in the ED to prevent hospitalization [10], earlier administration could have a greater effect on this important outcome. Administration early in the treatment course would better align with the NHLBI recommendation that IVMg be considered after the first hour of treatment [2]. Only 1 previous trial of IVMg with hospitalization as an outcome attempted to enroll children on arrival to the ED [11]. Before a large trial can determine the effect of early administration of IVMg on hospitalization, protocols to support the administration in the first 90 minutes of treatment should be tested and refined in a pilot trial setting.

Second, the safety of IVMg has not been well characterized in previous trials. Earlier small trials reported no significant adverse events (AEs) after IVMg but included too few children to conclude adverse effects were rare. In a study of 100 children who received a single dose of 50 mg/kg in an ED, 11 had hypotension after IVMg, though only 7 received any intervention, limited to an intravenous (IV) fluid bolus with normalization of blood pressure [12]. In a multinational survey of ED physicians, 24% reported hesitancy about using IVMg in children because of the risk of hypotension [10]. Collection of safety outcomes in a trial environment, including data collected after discharge from the ED, will allow enhancement and refinement of safety monitoring procedures for the future trial.

Third, the optimal dosage of IVMg in pediatric acute asthma is unknown [8]. Our current pharmacologic understanding of IVMg is insufficient to define which doses should be tested in clinical trials to carefully strike a balance between avoiding potential adverse effects and exploring maximal clinical effect. To safely explore thresholds of efficacy and safety in a large trial, we must fill gaps in the pharmacologic understanding of IVMg. To define the optimal dose of IVMg, safety and efficacy outcomes must be correlated with serum concentrations of magnesium in children receiving a range of IVMg doses.

Informative pharmacodynamic (PD) analysis requires a marker of respiratory status that can be measured reliably and repeatedly in all patients. The use of spirometry as an inclusion criterion in most previous trials [13,14] excludes most older patients in severe respiratory distress and also excludes children 4 years and younger by design, as they can rarely cooperate sufficiently to perform adequate spirometry. The Pediatric Respiratory Assessment Measure (PRAM; Table 1) is a clinical respiratory score that has been validated in children to be predictive of hospitalization [15,16] and to show a change in respiratory status after a bronchodilator [17]. A respiratory score such as the PRAM is valuable because it allows repeated measurement in every patient, allows comparison of respiratory status between treatment arms, and can serve as a measure of respiratory status for PD modeling.

**Table 1:** Pediatric respiratory assessment measure.

Score	Suprasternal retractions	Scalene muscle contraction	Air entry	Wheezing	O_2_ saturation, %
0	Absent	Absent	Normal	Absent	≥95
1	N/A^a^	N/A	Decreased at bases	Expiratory only	92-94
2	Present	Present	Widespread decrease	Inspiratory and expiratory	<92
3	N/A	N/A	Absent or minimal	Audible without stethoscope or silent chest with minimal air entry	N/A

^a^N/A: not applicable.

### Objectives

The objectives of this study are to (1) demonstrate the feasibility of enrolling children in the ED with severe acute asthma in a multicenter, randomized controlled trial (RCT) of a placebo, low-dose IVMg, or high-dose IVMg; (2) demonstrate the feasibility of timely delivery of study medication to enrolled patients and assessment of blood pressure and associated AEs in a standardized protocol; and (3) externally validate a previously constructed pharmacokinetic (PK) model [18] and develop a combined PK/PD model for IVMg using magnesium (total and ionized) serum concentrations and their correlation with measures of efficacy (improvement in respiratory distress) and safety in children with severe acute asthma.

## Methods

### Study Overview

This pilot trial is a prospective double-blind, placebo-controlled trial of children 2-17 years of age with severe acute asthma. It was endorsed by the Pediatric Emergency Care Applied Research Network (PECARN) and is being conducted at 3 PECARN sites in Utah, Pennsylvania, and Ohio. The goal is to evaluate study procedures and gather the data necessary to plan a larger trial.

### Study Population

Eligible participants are identified among children presenting to the ED for treatment of acute asthma. Specific criteria are listed in Textbox 1.

**Inclusion and exclusion criteria.**

**Inclusion criteria:**

- 2-17 years old
- Emergency Severity Index Triage acuity of 1 or 2
- Albuterol ordered to be administered in the emergency department (ED)
- A previous physician diagnosis of asthma confirmed by a licensed independent practitioner (ED attending, fellow physician, nurse practitioner, or physician assistant) in the ED who has spoken with the patient and family and reviewed the medical record
- Severe acute asthma, defined as a PRAM (Pediatric Respiratory Assessment Measure) score of 7 or greater as assessed by a treating physician at the time of screening using the study scoring instrument

**Exclusion criteria:**

- Positive pregnancy test in females of child-bearing potential (performed in all female potential participants 12 years and older) or known pregnancy (by a patient or parent report)
- Age-adjusted hypotension at presentation using age-based Pediatric Advanced Life Support parameters for systolic blood pressure in millimeters of mercury (children 1-10 years of age, systolic blood pressure (70 + 2×age in years) mm Hg; >10 years, systolic blood pressure <90 mm Hg) [19]
- Application of assisted ventilation before enrollment assessment (intubated, bilevel positive airway pressure, continuous positive airway pressure)
- Received IVMg within 24 hours before screening (by a parent, patient report, or medical record review)
- Enrollment assessment is 60 minutes or more after the start of ED treatment (start of first albuterol treatment)
- Previous enrollment in the same trial (by research staff review of trial records)
- Known severe renal impairment (by parent or patient report)

### Study Procedures

#### Overview

Descriptions of protocol procedures in this paper reflect protocol specifications and do not reflect whether protocol deviations have occurred. Significant protocol deviations will be reported in future publications.

#### Screening Schedule

Research staff identifies children who present to the ED with acute asthma during screening hours maintained by each study site.

#### Screening and Enrollment

**Figure 1:**
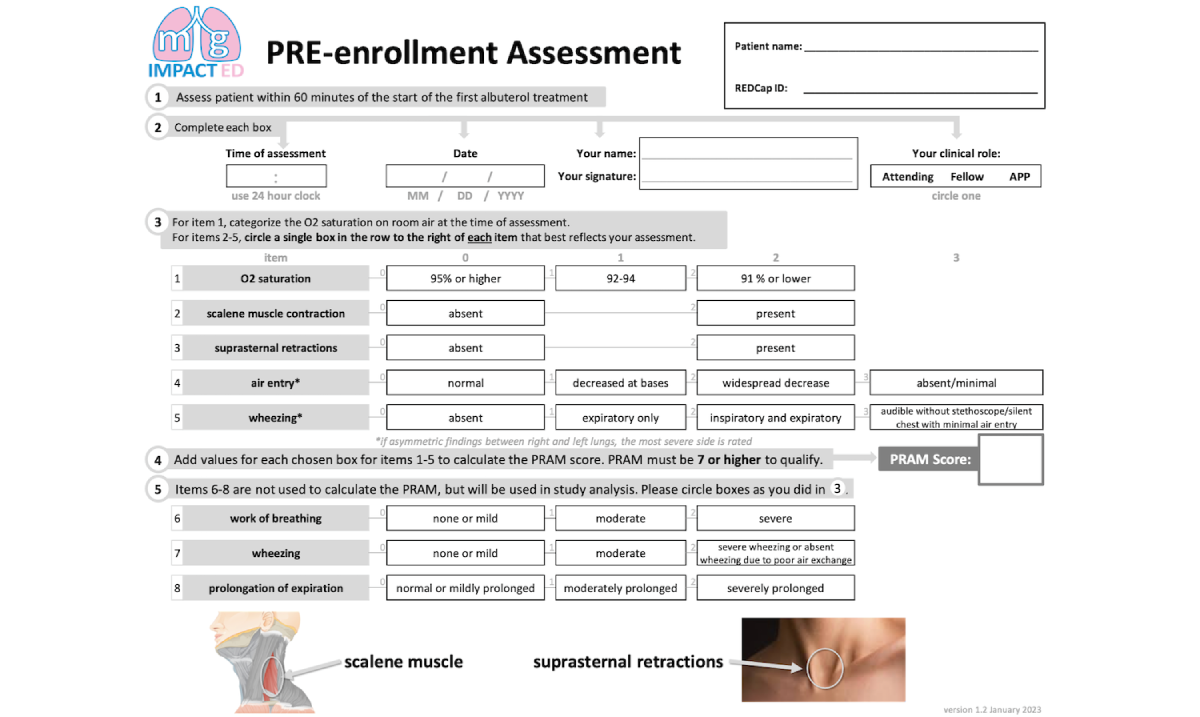
Preenrollment assessment including PRAM (Pediatric Respiratory Assessment Measure) score and items for pediatric asthma severity score [21].

Research staff identifies children within the study age range who have physician orders to receive inhaled albuterol and are triaged at an Emergency Severity Index [20] acuity of 1 or 2. The research staff member approaches the treating physician to determine if the child is being treated for asthma, and if the child meets other inclusion criteria. The physician examines the child and completes an asthma scoring instrument that generates the PRAM score (Figure 1). If the PRAM is high enough, research staff review exclusions with the clinician, and if no exclusions apply, they introduce the study to the patient and family.

**Figure 2:**
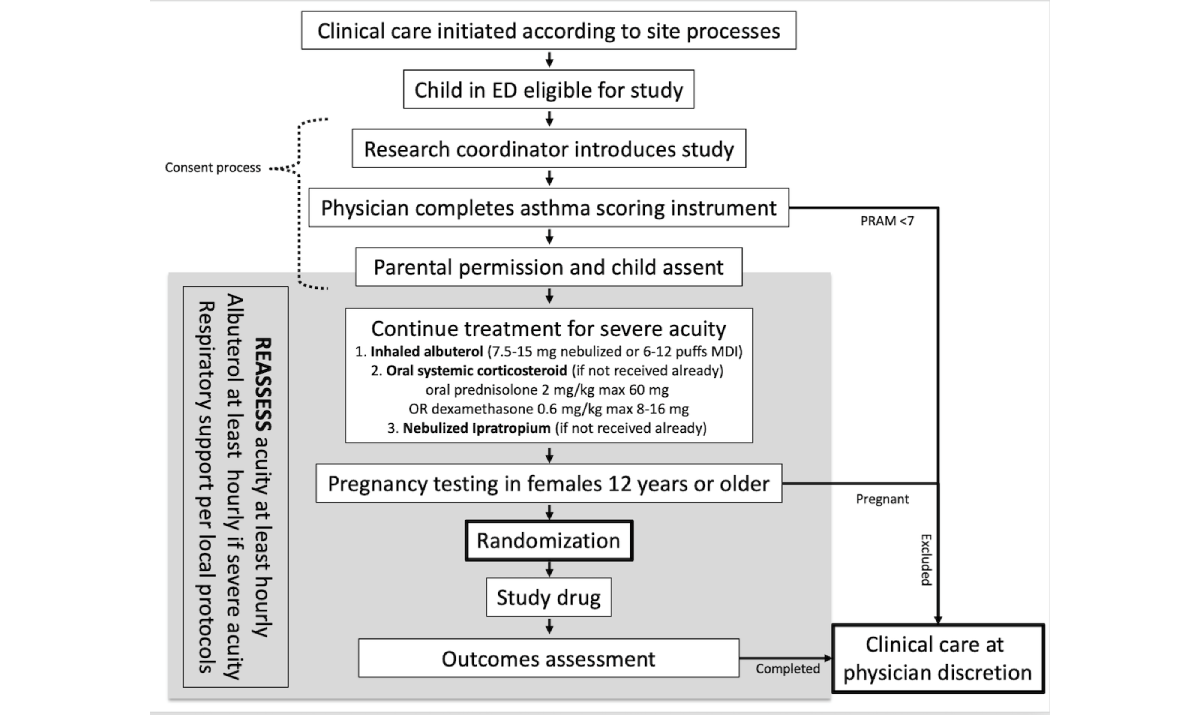
Study asthma treatment protocol. ED: emergency department; MDI: metered dose inhaler; PRAM: Pediatric Respiratory Assessment Measure.

If the PRAM score is 7 or higher, the research staff approaches the family for consent (including parental permission for all children and child assent for children 7 years and older) and enrollment. Consent documents are prepared in Spanish and English, but patients whose primary language (or the language of their guardian) is not English or Spanish are included if a professional interpreter can communicate the content of consent documents to the family and discuss study consent. After consent is complete, the child receives standard asthma treatment according to the study protocol (Figure 2). After consent, females 12 years and older without a reported pregnancy provide a blood or urine sample for qualitative pregnancy testing and are excluded from receiving the study drug if positive. Clinical staff reports pregnancy results to patients and families following institutional policies and state regulations.

#### Randomization

The investigational pharmacy at each institution prepares doses in a manner to maintain allocation concealment for all ED staff. A sequential randomization scheme with random-sized blocks stratified by each site was prepared in advance by a study statistician and entered into a Research Electronic Data Capture (REDCap) [22] database. After informed consent is completed, the physician requests the study drug from the pharmacy staff member, who then prepares and delivers the assigned dose for administration. The subject is considered enrolled when the earlier of 2 possible events occurs, that is, the REDCap randomization module is invoked to generate a randomization code for the patient, which code will then be relayed to the pharmacy, or the pharmacy dispenses a study drug dose based on the sequential randomization scheme. Nursing staff administers the dose regardless of the time since the initiation of treatment.

### Study Drug Administration

#### Acquisition

The specific study agent used in this pilot trial is magnesium sulfate in water for injection, a sterile, nonpyrogenic solution of magnesium sulfate heptahydrate in water. The study agent is acquired by each hospital’s research pharmacy from a common manufacturer as a solution of magnesium sulfate in water at 80 mg/mL. This agent is the commercially available preparation of IVMg and is shipped directly from the manufacturer to each study hospital pharmacy.

#### Preparation, Storage, and Labeling

Doses for each IVMg arm are prepared in an identical manner by drawing a specified volume of IVMg from the commercial container using a sterile technique and mixing it in a syringe or a polyvinylchloride container with a specified volume of sterile water. For the 50 mg/kg arm, this is accomplished by mixing 25 mL of IVMg (80 mg/mL) with 15 mL of sterile water for a final concentration of 50 mg/mL and a volume of 40 mL. For the 75 mg/kg arm, this is accomplished by mixing 37.5 mL of IVMg (80 mg/mL) with 2.5 mL of sterile water for a final concentration of 75 mg/mL and a volume of 40 mL. For the placebo arm, 40 mL of 0.9% sodium chloride solution are drawn into a polyvinylchloride container identical in appearance to the containers used for the IVMg arms.

Each prepared dose is labeled according to the sequential randomization scheme and stored according to the local pharmacy procedure. Either the pharmacist draws equivolumetric dosages (1 mL/kg, with maximum of 40 mL) from the prepared vials, or the pharmacist delivers the full dose to the bedside study team with instructions for the volume to be delivered.

#### Dosing Arms

Enrolled subjects are randomized to 1 of 3 arms:

1. IVMg 75 mg/kg arm: 75 mg/kg (maximum 3 gm, 40 mL) infused over 20 minutes through a peripheral IV catheter
2. IVMg 50 mg/kg arm: 50 mg/kg (maximum 2 gm, 40 mL) infused over 20 minutes through a peripheral IV catheter
3. Placebo arm: 1 mL/kg (maximum 40 ml) of normal saline over 20 minutes through a peripheral IV catheter

#### Dose Modification for Potential Toxicity

Clinicians are not to administer IVMg to enrolled subjects outside of the study protocol until study outcomes are determined 2 hours after the start of the infusion. The half-life of IVMg is approximately 2 hours [18]. Repeated-dose protocols in an intensive care unit setting that gave as much as 125 mg/kg IVMg to children with asthma over 2 hours produced no hypotension or other serious adverse effects [23]. Because of this margin of safety, open-label IVMg 50 mg/kg can be administered safely 2 hours after the study infusion under strict study monitoring protocols without the need for unblinding.

#### Unblinding Procedures

If a patient’s asthma symptoms deteriorate before 2 hours, clinicians are instructed to assume that the subject received the highest dose of active study drug and that they can give IVMg at a dose as high as 50 mg/kg open-label as soon as 120 minutes after the start of initial study infusion without unblinding, which would result in a maximum of 125 mg/kg over 2 hours if the patient is in the 75 mg/kg arm. Other interventions are given as needed in response to the patient’s condition. If unblinding is considered necessary, allocation information can be obtained from the site pharmacist, and unblinding is considered a study protocol violation.

#### Discontinuation of Study Drug

If hypotension is measured and perfusion is impaired (see Data Collection section), study infusion is stopped, clinicians give a 20 mL/kg bolus (maximum 1 L) of isotonic IV fluid and provide other interventions at the clinician’s discretion. If perfusion is normal, study drug infusion is continued at the same rate.

#### Withdrawal From Study

Subjects are observed in the ED for a minimum of 2 hours after the start of study infusion unless they require transfer to the intensive care unit due to escalation of therapy. Parents can withdraw permission for their children to continue in the study at any time. If a parent or child chooses not to continue in the study during study infusion, the infusion of the study drug is stopped. All patients who withdraw are followed for AEs and other symptoms.

### Data Collection

#### Baseline Data

**Figure 3:**
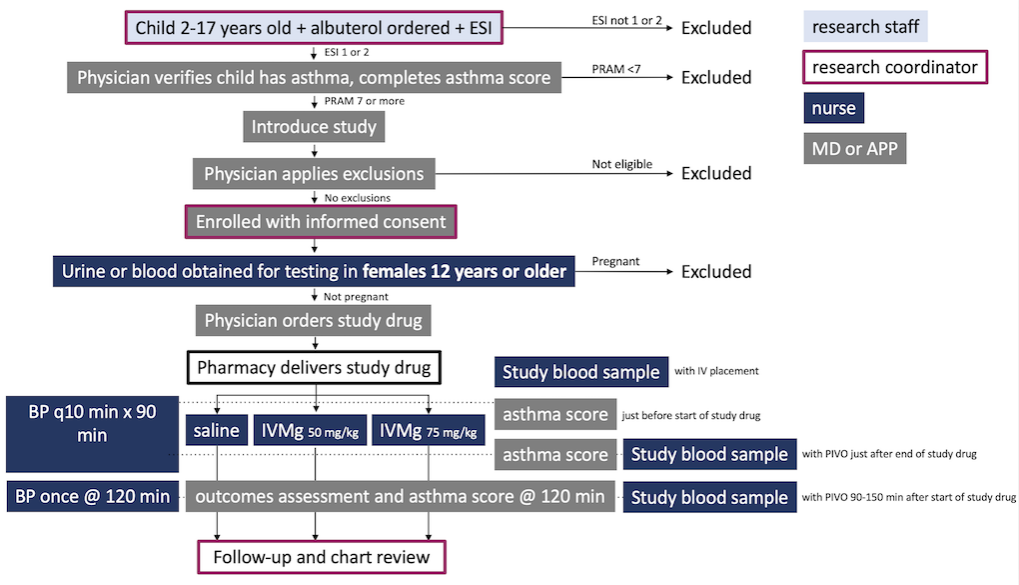
Schedule of Activities. APP: Advanced Practice Provider; BP: blood pressure; ESI: Emergency Severity Index; IVMg: intravenous magnesium sulfate; MD: physician; PRAM: Pediatric Respiratory Assessment Measure.

The research staff who approaches the patient for enrollment gathers baseline information from the child’s parent or guardian, including age, sex, race, ethnicity, chronic asthma control status and medications, treatments received for the current illness before ED presentation, and chronic medical conditions. The research staff member also collects data from the treating ED team, including the following items:

- Scoring: The treating physician completes a clinical asthma scoring instrument that includes the components of the PRAM immediately before, 20-40 minutes after, and 2 hours after the start of study infusion.
- Blood samples: They are drawn by clinical staff through the IV before the start of study infusion, then through the IV using a PIVO blood-drawing device (BD Medical) 20-40 minutes and 90-150 minutes after the start of study infusion. When the blood draw is not possible using PIVO, blood is drawn through the existing peripheral IV if possible.
- Blood pressure: It is measured by the study staff using an automated device just before the start of study infusion (within 10 minutes before the start of infusion), every 10 minutes (±4 minutes) for 90 minutes following the start of infusion, and once more 2 hours (±10 minutes) after the start of study infusion.
- Perfusion: If age-adjusted hypotension is detected, a treating clinician, which could be the treating nurse, completes a standardized assessment of perfusion based on World Health Organization criteria [24]. This assessment is then reviewed with the research staff. If perfusion is impaired during study drug infusion, study drug infusion is stopped.
- Timing: The research staff records time points of screening, identification, consent, and randomization, as well as times of infusion and blood draws, using the nurse’s documentation.
- Disposition: The research staff approaches the treating physician 2 hours after the start of study infusion and collects the stated disposition for the patient (hospitalization, discharge home, or uncertain), at which point study procedures are finished for the ED visit (see Figure 3).

Plasma is separated by centrifugation, transferred to a separate vial, stored at -80 C, and shipped to the study laboratory at the University of California, Davis

#### Follow-Up Assessments

For patients discharged to home, study staff calls or texts the family in 12-48 hours and in 1 week (5-10 days) to determine if they returned to any ED, the outcomes of any return visit, and to assess for AEs. All data points initially recorded on paper forms are securely stored and then entered into a REDCap database created by the Emergency Medical Services for Children Data Center (EDC) at the University of Utah.

#### Medical Record Review

Study staff reviews the electronic medical records of all subjects 1 week after enrollment to determine the actual patient disposition and hospital course.

#### Instruments

The PRAM is outlined in Table 1 and section 3 of Figure 1.

### Data Analysis

#### Specific Aim Analyses

##### Specific Aim 1

Demonstrate the feasibility of enrolling children in the ED with severe acute asthma in a multicenter RCT of a placebo, low-dose IVMg, or high-dose IVMg.

For the primary study hypothesis, we will identify, consent, and enroll 90 patients in 7 months or less. Our experience with previous trials within PECARN suggests a 7-month enrollment period is adequate to reach a stable rate of enrollment and demonstrate trial procedures. Because the future large RCT will need multiple sites for adequate enrollment, this pilot trial is testing trial procedures at 3 sites with various patient volumes, research infrastructures, and patient populations. Based on the projected 8% of asthma visits in the PECARN Registry that would qualify based on previous volumes at study sites, we anticipated that approximately 360 patients would qualify for the study during the 7-month study period, allowing for study completion even if only 25% of qualifying patients were enrolled. The study is expected to end once 90 patients are enrolled, even if this occurs in less than 7 months, but will not enroll for more than 9 months. An enrollment rate of 90 patients in 7 months for 3 sites would be sufficient to conduct a future trial powered to detect a difference in hospitalization, enrolling 800 patients in 23 months at 10 sites. A slower rate will prompt an adjustment of future trial length.

##### Specific Aim 2

Demonstrate the feasibility of timely delivery of study medication to enrolled patients and assessment of blood pressure and associated AEs in a standardized protocol.

Our first objective in this aim is to assess our ability to administer the study drug within 90 minutes after the start of asthma treatment. Timely delivery of study drug is considered feasible with current study procedures if 90% of study subjects received study medication within this time frame, giving time for study intervention to affect hospitalization and making hospitalization an outcome appropriate for the future trial. Delivery of study drug to less than 90% of study subjects indicates a need to further refine study procedures before the larger trial. We will optimize the delivery of study drug in the future trial by examining the impact of factors in the pilot trial that may influence study drug delivery. Factors associated with time to first study drug administration, administratively censored at 180 minutes, will be explored using Cox proportional hazards modeling. The Cox models are intended to identify features requiring additional planning to promote timely delivery rather than to find statistical significance.

Our second objective in this aim is to inform a safety monitoring plan for the future trial that is focused, clinically feasible, and effectively protects patient safety. We intensively monitor blood pressure (every 10 minutes) during the pilot trial for 90 minutes after the start of study drug infusion (see Data Collection section) and record all AEs from the time of randomization until the follow-up call approximately 1 week after enrollment. All serious, unexpected, and related AEs that are unresolved at the time of the patient’s termination from the study or discharge from the hospital will be followed by the Clinical Center investigators until the events are resolved, the subject is lost to follow-up, the AE is otherwise explained or has stabilized, or 12 months have passed from the time of last study dose.

We consider the procedures of the pilot trial feasible for the larger trial if we can collect 90% or more of planned data points related to blood pressure. This collection rate would indicate that this data gathering does not pose an undue burden on the teams providing clinical care to study patients. We will use the degree to which data points are collected in coordination with the frequency and timing of blood pressure changes, the frequency and degree of age-adjusted hypotension and poor perfusion, interventions delivered to patients with measured hypotension, and other recorded adverse effects to devise a monitoring plan for the future trial that captures all AEs and is maximally feasible.

##### Specific Aim 3

Externally validate a previously constructed PK model and develop a combined PK/PD model for IVMg using magnesium (total and ionized) serum concentrations and their correlation with measures of efficacy (respiratory distress) and safety (adverse effects) in children with asthma.

We will use these data to validate a PK/PD model that will guide dosing for the large trial. Ideal external validation of our previous PK model requires blood samples from a minimum of 30 patients who receive IVMg at sites outside of the site used to derive the PK model [18]. For an enrollment of 90 subjects, we anticipate approximately 40 subjects to meet this criterion. Data from the samples collected in this study will be used to externally validate our previously developed population PK model and, if needed (ie, median prediction error >15% or <−15%; median absolute prediction error >30%, calculated as the median of the values obtained from equations 2 and 3 for all collected samples), update the model with this larger sample size. Concentration analysis, PK model validation, and updating our previous PK model (if needed) will use similar approaches as we have previously described [25]. Notably, this includes accounting for endogenous magnesium concentrations (Equation 1) assumed to be at steady-state before IVMg administration.



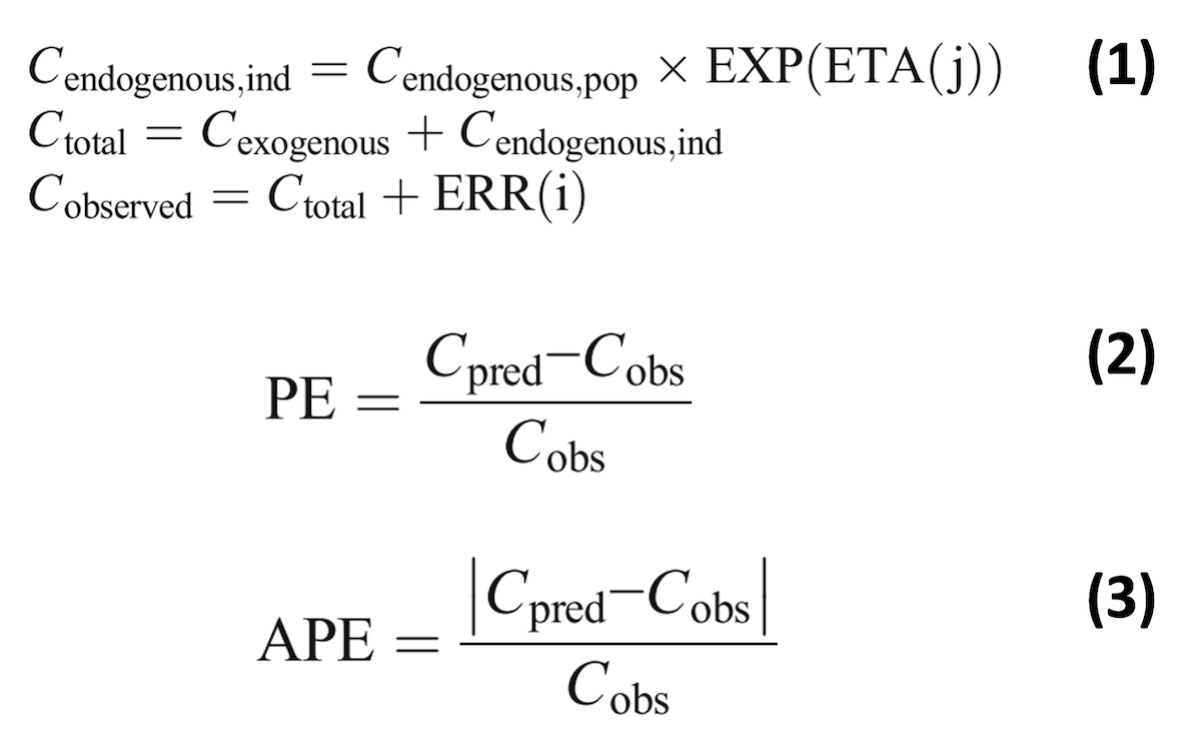



Once the PK model has been confirmed (or refined, if necessary), we will incorporate PRAM scores and other measures of respiratory distress to develop a population PK/PD model describing the relationship between IVMg systemic exposure and clinical response. Additionally, we seek to construct a PK/PD model describing the association between IVMg systemic exposure and AEs, including hypotension. PK/PD modeling will use NONMEM software (version 7.3, ICON Development Software). The first-order conditional estimation with the interaction method will be used throughout model building and evaluation. Model selection will be based on parsimony, objective function value (OFV), and visual diagnostic plots. Direct and indirect approaches to incorporating PD will be tested. After determining the base model, covariates will be tested for impact on PD using a forward inclusion (*P*<.05) and backward exclusion (*P*<.01) method. Covariates will be added to the model in a stepwise fashion and allowed to remain in the model if covariate inclusion decreases the OFV by at least 3.84 and its exclusion increases the OFV by at least 6.63. Once the final PK/PD model is determined, we will evaluate model accuracy and robustness through bootstrapping methods and visual predictive checks.

Dose-exposure response data simulated (1000 data sets) from the PK/PD model will be used to select doses used in the future large trial. Specifically, we will use the simulated data sets to determine the dose at which 10% of participants are expected to have adverse effects (ie, hypotension) and establish an upper limit of dosing for the future trial. Importantly, this analysis will allow us to evaluate the role of an upper dose limit (ie, the 2 or 3 gm maximum dose, regardless of patient weight) in preventing hypotension. Similarly, we will use the simulated PK and PD data to define doses expected to yield 20%, 40%, and 60% reductions in PRAM and hospitalization, respectively. We plan to target the use of these doses in the future study as the doses that are expected to maximally reduce hospitalization.

If we are unable to build a robust population PK/PD model using NONMEM, we will evaluate correlations between PK and PD using Pearson correlation analyses in GraphPad Prism version 6.05 (or later) software. In this scenario, the population PK model will be used to identify doses that generate simulated concentration data within the target therapeutic range established by the correlation analyses.

Per the PECARN Registry, approximately one-fourth of children receiving IVMg for asthma weigh more than 40 kg, the weight at which a 2 gm (50 mg/kg) or 3 gm (75 mg/kg) dose is reached. As a result of this maximum, children weighing more than 80 kg (low-dose arm) or 120 kg (high-dose arm) would implicitly have an intended dose less than 25 mg/kg IVMg, below the lower end of the range of doses in the guidelines. As a drug that exhibits first-order kinetics, delivering IVMg with an arbitrary maximum may reduce the benefit of the drug in a substantial portion of children. We will use the newly developed PD model and validated PK model to determine if patients above 40 kg have less improvement in clinical score and if any associations found are related to changes in serum values.

#### Sample Size Calculations and Statistical Power

Sample size for this pilot trial was driven by the first aim, which is to demonstrate the ability to enroll children severely ill with asthma in an RCT. The second aim is focused on the feasibility of study drug delivery and the collection of safety data, and the third aim is focused on pharmacologic modeling for dose estimation to be used in the large trial to follow this pilot trial. All 3 aims of the pilot trial are focused on generating data to inform the structure of the larger trial to follow, and accordingly, they do not have sample size calculations or associated calculations of statistical power.

### Protection of Human Subjects

#### Ethics Approval

A single investigational review board (IRB) was used for this study. Study sites relied on the University of Utah IRB to act as the IRB of record (IRB #00104082). The EDC and each clinical center obtained IRB approval before participating in the study.

#### Risk Benefit Assessment

Asthma is a leading cause of hospitalization in children and incurs substantial costs for the health care system [1]. Previous trials of IVMg in children with asthma were too small to conclude whether IVMg can reduce hospitalization in children with asthma. To date, these small trials of IVMg in acute asthma have not been followed by larger, conclusive trials. Comparison to placebo in a large trial is needed to evaluate the effect of IVMg on hospitalization.

Risks of IVMg have been described in previous literature but not sufficiently to dismiss them as minor. This study will help ensure that monitoring for children enrolled in future trials is feasible and complete.

Considering the potential benefits and the anticipated risks, the research outlined in this proposal will provide important evidence to guide the care of children with asthma. By clarifying the efficacy, safety, and dose of IVMg for children with asthma, these results will influence the care provided to severely ill children with asthma nationally and globally through inclusion in NHLBI guidelines and other guidelines such as those developed by the Global Initiative for Asthma.

#### Population to Be Studied

The enrollment of patients in this trial is expected to reflect the population of children with severe acute asthma at each of the enrolling sites with respect to age, sex, race, and ethnicity.

### Data and Safety Monitoring Plan

#### Data Safety Monitoring Board (DSMB)

Each clinician caring for a study patient had primary responsibility for the safety of the individual subjects under his or her care. A DSMB was established before the initiation of enrollment. The DSMB consisted of 3 members, 1 of whom had statistical expertise and 2 of whom had experience in emergency medicine. All individuals had experience in the conduct of randomized clinical trials and had no direct association with the clinical sites in the trial or other conflicts of interest that would interfere with their objectivity. We did not anticipate having sufficient interim data during the trial for the DSMB to reach conclusions regarding efficacy or safety, though safety data were considered in interim analysis. The DSMB is responsible for approval of the final study design, reviewing and analyzing the progress of the study, monitoring the safety of the study treatments (as outlined below), and reviewing reports of data quality. The DSMB met before the first site was activated for enrollment and again after approximately 30 subjects were enrolled. The DSMB, or study principal investigator (PI), had the power to request additional meetings. The DSMB has the power to halt the study if safety reasons exist.

#### Study-Specific AE definitions

Hypotension is defined as any systolic blood pressure (mm Hg) meeting the following age-specific norms [19]:

1. Children 1-10 years: <70 + (age in years × 2)
2. Children >10 years: <90

Measured hypotension without associated symptoms is categorized as a mild AE; the presence of symptoms is categorized appropriately based on the degree of impairment of activity.

A serious AE is defined as any experience that results in death, is life-threatening, results in prolongation of existing hospitalization, results in a permanent disability, results in congenital anomaly/birth defect, or any other event that, based upon appropriate medical judgment, may jeopardize the subject’s health and may require medical or surgical intervention to prevent one of the other outcomes listed in this definition.

#### Investigational New Drug Application Information

Investigation of IVMg in this study is supported and supervised by the Food and Drug Administration (FDA) through Investigational New Drug Application No. 133781.

#### Study Training

A formal training program for investigators and research staff was held before the start of enrollment. The training covered regulatory topics, including applicable drug regulations and training in good clinical practice. The training provided in-depth explanations regarding study procedures, clinical care, AE reporting, data entry procedures, quality assurance, site monitoring, and the informed consent process. A manual of study operations was created and provided to each site investigator before the start of enrollment. The manual detailed specific information about the study procedures, regulatory information, safety reporting, and other necessary information. Updates and revisions to the manual were made available electronically. The EDC, in collaboration with the study PIs and clinical coinvestigator (MDJ, JJZ, and Dr Shihabuddin), was the main contact for study-related questions.

Before the start of enrollment, lead investigators and each site’s research coordinator, along with EDC staff, met virtually or in person for a training session. Each site investigator instructed the ED physicians at their home institutions about the study procedures, provided training on the PRAM score, served as a local advocate and champion for the study, and answered questions as they arose. Throughout the study, the study investigators and research coordinators have at least monthly scheduled telephone conference calls or webinars for all study personnel and more frequent group communications, as necessary. BD Medical conducted training on the use of PIVO with blood-collection staff before subject enrollment at each site.

Monitoring of the research activities conducted during the pilot trial is supported by quality assurance visits conducted regularly by the nodal administrators of the involved PECARN sites, including regular assessment of support and funding for study effort and preparation for monitoring by the EDC and other data regulatory bodies, including the Food and Drug Administration. Each quality assurance visit is followed by a joint discussion with the site PI and study staff to define isolated, structural, or study-related barriers to study success.

#### Public Use Data Set

After subject enrollment and follow-up have been completed, the EDC will prepare a final study database for analysis. A releasable database will be produced and completely deidentified in accordance with the definitions provided in the Health Insurance Portability and Accountability Act (HIPAA). Namely, all identifiers specified in HIPAA will be recorded in a manner that will make it impossible to deduce or impute the specific identity of any patient. The database will not contain any institutional identifiers.

The EDC will also prepare a data dictionary that provides a concise definition of every data element included in the database. If specific data elements have idiosyncrasies that might affect interpretation or analysis, this will be discussed in the dictionary document. In accordance with policies determined by the investigators and funding sponsors, the releasable database will be provided to users in electronic form.

## Results

The project was funded in March 2022, began enrollment in September 2022, will stop enrollment no later than the end of May 2023, and we expect to submit results for publication in late 2023.

## Discussion

### Overview

The study design for this pilot trial mirrors the design that we expect to use in the larger, conclusive trial. The future trial will be powered to evaluate the effect of bolus IVMg on hospitalization and will be conducted at more sites, while the pilot trial is designed to demonstrate feasibility and collect safety and pharmacologic data needed to optimize the design of the future trial. In this pilot trial, we seek to shift current research paradigms regarding the application of pharmacologic principles to pediatric clinical trials. More pharmacologic rigor in trials which include children would result in improved pharmacotherapy for children. In this project, we incorporated 3 key related innovations that improve our ability to integrate pharmacologic principles into the future large trial, and pediatric clinical trials in general.

First, we implement improved collection of study blood samples with less pain using a clinical device, which circumvents the need for repeated venipuncture for blood samples. Medication dosages used in children generally rely on prescribing data derived from adults, in small trials, or by group consensus [26]. Even when medications are tested in children in a trial, inadequate consideration of dosing is the most common reason a trial fails [27]. Defining appropriate pediatric doses within a trial requires repeated serum samples. Historically these samples were collected using repeat venipuncture to measure serum concentrations to inform PK models. This was how samples were collected during routine clinical care to inform the model we previously constructed using retrospective clinical data [18]. Pain and distress are the most common reasons for a child to reject participation in a trial, and children commonly experience pain and distress during venipuncture [28]. As a result, many studies do not collect the PK data needed to define dosing, and most previous trials of IVMg in children did not obtain serum samples. The 3 trials that obtained samples obtained only 2 per subject [13,14,29], insufficient for robust pharmacologic analysis or modeling. This current state of dose planning in trials involving children presents a pressing need to identify innovative strategies to collect serum samples from children while avoiding the pain and distress of venipuncture. The PIVO device is currently used clinically to facilitate repeat blood draws without repeat venipuncture, but it is not widely used in clinical research aside from a prospective study we conducted to validate our PK model [25]. As expected from our previous single-site study, we expect that use of the PIVO device at all sites in the pilot trial will reduce harm to children who participate in the trial, increase acceptability to children and parents (35/50, 70% of eligible children were enrolled in our previous study), and result in higher sample collection rates than expected with repeat venipuncture or drawing blood from the existing IV (29/35, 83% with 3 samples in our previous study using PIVO vs 97/308, 31% in previous PECARN studies).

Second, we incorporate trial optimization and dose finding in pediatric asthma guided by PK/PD modeling. Previous trials of IVMg in children with asthma in the ED did not use pharmacologic principles to select an optimal dose for evaluation. Though pharmacologic principles have been used to select dosages for many trials in children and adults, our application of PK and PD methods to the study of pediatric asthma in the ED is novel. Our team’s preliminary studies in the ED demonstrated our ability to collect and analyze pharmacologic data, and we used these data to select doses for the pilot trial with maximal rigor. We will use the PIVO device to enable the collection of repeat serum samples, and we will use the data from our samples to integrate and refine our PK and PD models to optimize the doses we select for the future large conclusive trial.

Third, we evaluate early administration of intensive asthma treatment in a trial setting*.* Previous trials did not attempt to deliver the study drug to children with asthma early in the ED treatment. This reflects an outdated clinical paradigm of pediatric acute asthma treatment based on administering intensive treatments only when initial treatments have been ineffective. Though this is generally how care delivery is outlined in clinical guidelines published in 2007 [2], subsequent evidence supports the administration of continuous beta-agonists [30], steroids [31], and IVMg [32] *as early as possible for patients with severe illness*. Improving the timeliness of treatment delivery is a major focus of quality improvement in the ED care of children with asthma [33,34]. Early administration of intensive treatment to children with asthma in this project is a novel application of high-quality clinical care delivery in a trial setting.

Previous trials of IVMg are insufficient to guide the clinical use of IVMg. The equipoise needed to study the effect of IVMg versus placebo on hospitalization in a large clinical trial is illustrated by a combination of possible but uncertain clinical benefits for ill children, variation in clinical use, lack of consensus regarding clinical benefit, and evidence gaps from previous trials. IVMg is inexpensive, readily available, and widely used in current clinical settings, but it lacks evidence to guide its current use for children with asthma.

### Limitations

A large trial in children severely ill with acute asthma, like other clinical trials in acutely ill children, is at risk of low patient accrual if not properly planned and conducted [26], including the risk that physicians may hesitate to enroll patients being randomized to either study therapy or placebo. With this trial, we will further the previous expertise of PECARN in conducting research on severely ill children. The trial outlined in this proposal represents a novel challenge for this network by requiring rapid enrollment after patient arrival, providing an opportunity to understand and manage these risks and challenges in the future trial.

Parental consent may be a barrier to enrollment, although consent in previous trials of children with acute respiratory illness conducted in PECARN has been obtained at rates of approximately 50% [35]. This pilot trial is structured to allow enrollment of our sample size during our 7-month study window even if only 25% of the estimated number of eligible patients consented and enrolled, though the number of patients eligible for enrollment could differ due to episodic variations in asthma illness that cannot be controlled or anticipated.

This pilot study anticipates 3 blood draws per patient: 1 with IV placement and 2 from the IV after infusion of study drug. Repeat blood draws from an IV avoid additional venipuncture but are at risk for failure or loss of the IV line, which would result in fewer than anticipated serum measurements. In a previous study of IV benzodiazepines conducted in PECARN [36], 64% (197/308) of patients successfully had multiple blood draws from a single IV (internal data). During preliminary study at 1 of the study sites [25], the PIVO vascular access device for IV blood draws allowed 2 consecutive blood draws from a peripheral IV in 90% of patients and 3 consecutive blood draws in 83%, which was adequate for trial procedures.

### Conclusions

IVMg has the potential to improve the outcomes of ED treatment for children with severe acute asthma but requires further evaluation of its efficacy, safety, and optimal dosage in a robust pediatric trial setting. More broad and standardized use of IVMg, if effective, may reduce hospitalizations and related health care costs for children and would be cost-effective. An economic analysis of children with asthma at 2 hospitals concluded that the use of IVMg reduced costs by 28% and increased quality-adjusted life years [37]. If IVMg is found to not be effective in a well-powered clinical trial, its use could be appropriately guided and restricted by the trial’s results, future investigations can focus on other therapies, and these findings should be included in institutional and national asthma guidelines. This pilot trial will provide important information to guide a larger, conclusive trial that can fully evaluate the effect of IVMg on hospitalization of children with severe acute asthma.

## Appendix

Multimedia Appendix 1 Most recent peer review of protocol by National Heart, Lung, and Blood Institute.

## Abbreviations

AE: adverse event
DSMB: Data Safety Monitoring Board
ED: emergency department
EDC: Emergency Medical Services for Children Data Center
HIPAA: Health Insurance Portability and Accountability Act
IRB: investigational review board
IV: intravenous
IVMg: intravenous magnesium sulfate
NHLBI: National Heart, Lung, and Blood Institute
OFV: objective function value
PD: pharmacodynamic
PECARN: Pediatric Emergency Care Applied Research Network
PI: principal investigator
PK: pharmacokinetic
PRAM: Pediatric Respiratory Assessment Measure
RCT: randomized controlled trial
REDCap: Research Electronic Data Capture

## Data Availability

Within 6 months of final patient contact and within 3 months of data lock, the data sets generated and analyzed during this study will be prepared by the EDC as a deidentified database accessible for public use [38].
