# Supplementary material for: Intravenous Magnesium: Prompt Use for Asthma in Children Treated in the Emergency Department (IMPACT-ED): Protocol for a Multicenter Pilot Randomized Controlled Trial
Source: JMIR Res Protoc. 2023 Jul 17;12:e48302. doi: 10.2196/48302 (PMC10391520; doi:10.2196/48302)
Supplement: Multimedia Appendix 1 [file resprot_v12i1e48302_app1.pdf]

SUMMARY STATEMENT
(Privileged Communication)

PROGRAM CONTACT:
Michelle Freemer
301-435-0202
michelle.freemer@nih.gov

Release Date: **07/09/2021**
Revised Date:

Application Number: **1 R34 HL152047-01A1**
Formerly: **1R34HL152047-01**

Principal Investigators (Listed Alphabetically):

JOHNSON, MICHAEL DAVID (Contact)
ZORC, JOSEPH J

Applicant Organization: UNIVERSITY OF UTAH

Review Group: **SSPT (OA)**
NHLBI Single-Site and Pilot Clinical Trials Study Section

Meeting Date: **06/23/2021**
Council: **OCT 2021**
Requested Start: **12/01/2021**
RFA/PA: **PAR21-079**
PCC: **LLAG N**

Project Title: **Intravenous Magnesium: Prompt Use for Asthma in Children Treated in the Emergency Department (IMPACT-ED)**
SRG Action: **Impact Score:10**
Next Steps: **Visit https://grants.nih.gov/grants/next_steps.htm**
Human Subjects: 30-Human subjects involved - Certified, no SRG concerns
Animal Subjects: 10-No live vertebrate animals involved for competing appl.
Gender: 1A-Both genders, scientifically acceptable
Minority: 1A-Minorities and non-minorities, scientifically acceptable
Age: 2A-Only Children, scientifically acceptable

Project Year	Direct Costs Requested	Estimated Total Cost
1	225,000	366,769
2	225,000	366,769
TOTAL	450,000	733,538

ADMINISTRATIVE BUDGET NOTE: The budget shown is the requested budget and has not been adjusted to reflect any recommendations made by reviewers. If an award is planned, the costs will be calculated by Institute grants management staff based on the recommendations outlined below in the COMMITTEE BUDGET RECOMMENDATIONS section.

1R34HL152047-01A1 Johnson, Michael

RESUME AND SUMMARY OF DISCUSSION: This resubmission application proposes to conduct a pilot randomized 3-arm trial in N=90 eligible children with asthma in the emergency room comparing high dose IV magnesium, low dose IV magnesium, and placebo, as part of first line treatment within 90 minutes of nebulizer administration, with the primary endpoint of hospital admission. The trial will be conducted in three sites in the Pediatric Emergency Care Applied Research Network (PECARN). The rationale for the intervention is that some evidence supports the effectiveness of IV magnesium for acute asthma flare but the evidence is inconsistent, use varies, and a definitive trial is needed to inform practice. The significance of the problem addressed was judged to be high and the novelty also to be high in that a successful trial has the potential to change practice. Additional strengths identified in the discussion include the clinically relevant trial endpoint, the strong multi-disciplinary team including a well-developed MPI model and inclusion of a biostatistician and pharmacist, the research environment which includes the PECARN, the novelty and appropriateness of the PIVO device to draw blood without causing pain, the plan for an adaptive dose design in the planned larger trial, and sound data analysis and trial management plans. The application has been revised in response to previous critiques and is significantly improved. Minor weaknesses were also discussed, including that the pilot sample size is insufficient for the safety aim, although it was also noted that the plan to monitor for hypotension was well described, and the burden of the trial on the ED physicians and nurses was possibly underestimated. Overall impact was judged to be very high.

DESCRIPTION (provided by applicant): Asthma is the most common chronic illness of childhood and a leading cause of hospitalization and healthcare costs for children. Most children hospitalized for asthma first receive breathing treatments and steroid medicines in an emergency department (ED) according to national guidelines. An additional medicine, intravenous magnesium sulfate (IVMg), may help severely sick children avoid hospitalization. National asthma guidelines recommend IVMg for severely sick children, but note a lack of consistent evidence to support this recommendation. Only about one in four children hospitalized for asthma received IVMg in the ED. Estimates of the potential effects of broader use of IVMg on hospitalization are limited by the small size of prior trials, but increased use could potentially avoid 18,000 hospitalizations each year, producing direct cost savings of $65 million yearly in addition to saving significant indirect costs of missed school and parental work. A few major questions remain about IVMg. First, it has not been tested early in ED treatment, when the impact on hospitalization would be greatest. Second, the clinical impact of hypotension, a driver of low utilization, is not characterized in prior trials or clinical databases. Third, no trials have compared different IVMg doses or measured serum magnesium levels to optimize dosing, so the most effective dose is unknown. All prior trials of IVMg in children with asthma have been small. A large randomized, placebo-controlled clinical trial of IVMg that could answer whether IVMg can reduce hospitalization might be challenging for a few reasons. First, enrolling patients fast enough to give IVMg early in ED treatment is challenging. Second, understanding blood pressure changes after IVMg is essential to plan safety monitoring for a larger trial. Third, little pharmacologic information has been gathered to guide the doses of IVMg to be tested. We must conduct a small pilot clinical trial to test our procedures and gather necessary information to plan the large trial. The Pediatric Emergency Care Applied Research Network (PECARN) is a network of children's EDs that has conducted similar research involving acutely ill children. Our goals in this project are to: 1. Demonstrate the feasibility of enrolling children in the ED with severe acute asthma in a multicenter, randomized, controlled trial of placebo, low-dose IVMg, or high-dose IVMg. 2. Demonstrate the feasibility of timely delivery of study medication to enrolled patients and assessment of blood pressure and associated adverse events in a standardized protocol. 3. Externally validate a previously constructed PK model and develop a combined PK/PD model for IVMg using magnesium (total and ionized) serum concentrations and their correlation with measures of safety and respiratory distress in children with asthma. After completing this pilot trial in three sites, we will plan a multi-center randomized trial of IVMg in children with severe acute asthma at more sites to enroll enough children to know if IVMg can reduce hospitalization.

PUBLIC HEALTH RELEVANCE: Many children currently being hospitalized with severe asthma could potentially avoid hospitalization and be sent home if their treatment in the emergency department was more effective. We will conduct a pilot trial that will lead to a larger study to conclusively answer whether a simple and inexpensive medicine, intravenous magnesium sulfate, can be used in the emergency department to prevent hospitalization for these children.

CRITIQUE 1:

Significance: 2
Investigator(s): 1
Innovation: 2
Approach: 2
Environment: 1

Overall Impact: The investigators resubmit a proposal to conduct a feasibility, safety, and dose trial of IV magnesium as first line emergency department treatment (within 90 min) of severe acute asthma in 90 children ages 2-17 years. Primary outcome of proposed full-scale trial is hospitalization (proposed n=800). Pediatric asthma is important, common, and nationally resonant; advances in treatment have been limited. The premise for magnesium is supported by preliminary data but investigations in this population and setting are sparse. If shown effective, a large scale RCT could change guidelines and practice significantly. This pilot addresses feasibility of RCT enrollment, timely delivery, and development of a PK/PD model at 3 sites. Minor weaknesses identified in the initial submission included using a pilot trial to inappropriately address safety, age enrollment issues, lack of some detail in the mechanics of the approach, data management concerns, and some statistical issues. The investigators have been responsive to the original critiques, but a few minor issues remain. Overall, an excellent responsive resubmission, high significance, a few minor weaknesses. Impact judged to be outstanding.

1. Significance:
Strengths

- Addresses the most common chronic illness of childhood. It is a leading cause of hospitalization and is very costly. Inexpensive therapeutics used to possibly reduce hospitalizations are highly significant.
- Justification for a pilot trial is good – will allow for examining feasibility of early treatment and different doses.
- The phase adaptation planned for the possible future RCT is significant (see Innovation).
- NHLBI supports IVMg for this condition but notes lack of consistent evidence.
- Early administration may have the largest effect on outcomes.
- Excellent preliminary data supports the significance, although trials have been small.
- Equipoise exists illustrated by possible but uncertain clinical benefit for ill children, variation in use, lack of consensus regarding benefit, and evidence gaps from prior trials.
- The drug is inexpensive.
- Addresses first line therapy, not those refractory to initial treatment.
- Exceptionally well-written protocol.

Weaknesses

- Measuring preliminary safety is laudable and needs to be done, but pilot trials are underpowered to address. Overreliance in these measurements could be negative for patients

in the design of a larger trial. See: https://www.nccih.nih.gov/grants/pilot-studies-common-uses-and-misuses. The ability to make meaningful observations regarding hypotension is limited and a bit overstated in the proposal, albeit this is attenuated somewhat with the focus on monitoring procedures/plan as the pilot outcome.

- Would have been convenient to identify the major changes within the proposal itself.
- If Mg ultimately is shown to have weak efficacy, its use might increase costs and resources.

2. Investigator(s):

Strengths

- MPI model is appropriate. The co-PIs are pediatric emergency medicine physicians with collaborative research experience in this area.
- Successful track records within the emergency care environment.
- Broad expertise including biostatistics and pharmacology.

Weaknesses

- Relatively junior contact PI clinical trial experience, but great supporting cast and network.

3. Innovation:

Strengths

- The phase adaptation planned for the possible future RCT is innovative and allows for a reduction of subjects needed and minimizes patients randomized to disadvantageous arms.
- The needle free device is a nice addition.
- Very early first line administration is novel.

Weaknesses

- Medication is not novel, but it is inexpensive and widely available.
- PECARN is a fabulous resource but has been around for decades and is not novel.

4. Approach:

Strengths

- Much work is presented in preparation for this pilot.
- FDA IND approval letter provided.
- Planned analyses and statistical approach are appropriate.
- Data management is a strength.
- The design has a strong rationale.
- Accommodations are presented in approach for young children.
- DSMB plan adequate.

Weaknesses

- The ability to make meaningful observations regarding hypotension is limited and a bit overstated in the proposal, albeit this is attenuated somewhat with the focus on monitoring procedures/plan as the pilot outcome.

- Not entirely clear how the likely small #'s with transient hypotension will inform the future RCT monitor plan that is focused, effective, and clinically feasible. Not sure if "clarify concerns conclusively for clinicians" (page 83, top) refers to the pilot or future trial.

Study Design:

Strengths

- PECARN network is well established and productive. It is a major strength.
- The design is methodologically sound.
- Ethical issues addressed.

Weaknesses

- None noted.

Data Management and Statistical Analysis:

Strengths

- Information gained will likely be sufficient to decide about the subsequent trial. This process is clearly defined.

Weaknesses

- None noted.

5. Environment:

Strengths

- MPI model description is robust and strong, including conflict resolution.
- Overall, the environment is excellent.

Weaknesses

- The Structure of the Study Team document could have been improved with a figure illustrating relationships. Also the relationship with NHLBI and the DSMB.

Study Timeline:

Strengths

- Appropriate enrollment projections.
- Detailed, excellent timeline including training, start-up, quality control, monitoring, and analysis.

Weaknesses

- None.

Protections for Human Subjects:

Acceptable Risks and/or Adequate Protections

- Acceptable.

Data and Safety Monitoring Plan (Applicable for Clinical Trials Only):

Acceptable

- DSMB described and appropriate.

Inclusion Plans:

- Sex/Gender: Distribution justified scientifically
- Race/Ethnicity: Distribution justified scientifically
- For NIH-Defined Phase III trials, Plans for valid design and analysis: Not applicable
- Inclusion/Exclusion Based on Age: Distribution justified scientifically
- Acceptable.

Vertebrate Animals:

Not Applicable (No Vertebrate Animals)

Biohazards:

Not Applicable (No Biohazards)

Resubmission:

- Responsive, see above.

Applications from Foreign Organizations:

Not Applicable (No Foreign Organizations)

Select Agents:

Not Applicable (No Select Agents)

Resource Sharing Plans:

- Acceptable.

Authentication of Key Biological and/or Chemical Resources:

Not Applicable (No Relevant Resources)

Budget and Period of Support:

Recommend as Requested

Recommended budget modifications or possible overlap identified:

CRITIQUE 2:

Significance: 1
Investigator(s): 2
Innovation: 2
Approach: 2
Environment: 1

Overall Impact: This revised R34 application entitled “Intravenous Magnesium: Prompt use for asthma in children treated in the Emergency Department (IMPACT-ED)” aims to assess the utility of early IVMg in the treatment of asthma exacerbations in the ED in order to attempt to prevent hospitalizations in children. The problem is highly significant as a leading cause of hospitalization in children in the US. Treatments of acute asthma have not improved significantly in the last several decades beyond providing more timely and protocolized interventions. The proposal, if successfully completed would provide the information necessary to perform a large RCT within PECARN. The intervention is not particularly innovative but its use early in ED treatment is not common in most pediatric settings. Application provides evidence that lack of use of IVMg in ED driven by safety concerns such as hypotension, providing justification for this inquiry. ED intervention studies with acutely ill children are challenging, but PECARN has a strong track record of success.

1. Significance:
Strengths
- Acute wheezing is common and costly.
- Limited treatments available for this common ED condition.
- IVMg is inexpensive and readily available for implementation.

Weaknesses
- None noted.

2. Investigator(s):
Strengths
- PECARN network.
- Excellent team assembled across all aspects of study.

Weaknesses
- Contact PI relatively early career but MPI approach and overall study team strength mitigate this.

3. Innovation:
Strengths
- Early use IVMg rather than rescue after failure of early treatment in ED.
- Adaptive design to subsequent trial.

Weaknesses
- IVMg itself is not an innovative intervention.

4. Approach:
Strengths
- Early intervention feasibility well thought out and discussed, including opportunity for enhanced benefit.
- Need for safety assessments is better delineated in revised application – barrier to use currently.
- Subsequent adaptive dose study.

Weaknesses

- Burden on busy ED clinicians is not insignificant and not acknowledged.
- Lacks discussion of standardized training for PRAM assessments, which will need to occur across larger study.

Study Design:

Strengths

- Well thought out study design.

Weaknesses

- None noted.

Data Management and Statistical Analysis:

Strengths

- Clear definition of data management.
- Necessary and sufficient for subsequent trial.

Weaknesses

- Need standardized training for PRAM.

5. Environment:

Strengths

- Excellent environment at University of Utah Peds ER and with PECARN.

Weaknesses

- None.

Study Timeline:

Strengths

- Realistic timeline.

Weaknesses

- None noted.

Protections for Human Subjects:

Acceptable Risks and/or Adequate Protections

Data and Safety Monitoring Plan (Applicable for Clinical Trials Only):

Acceptable

Inclusion Plans:

- Sex/Gender: Distribution justified scientifically
- Race/Ethnicity: Distribution justified scientifically

- For NIH-Defined Phase III trials, Plans for valid design and analysis: Not applicable
- Inclusion/Exclusion Based on Age: Distribution justified scientifically
- Pediatric population justified.

Vertebrate Animals:
Not Applicable (No Vertebrate Animals)

Biohazards:
Not Applicable (No Biohazards)

Resubmission:
- Responsive to prior critiques.

Applications from Foreign Organizations:
Not Applicable (No Foreign Organizations)

Select Agents:
Not Applicable (No Select Agents)

Resource Sharing Plans:
Acceptable

Authentication of Key Biological and/or Chemical Resources:
Not Applicable (No Relevant Resources)

Budget and Period of Support:
Recommend as Requested
Recommended budget modifications or possible overlap identified:

CRITIQUE 3:

Significance: 1
Investigator(s): 1
Innovation: 1
Approach: 1
Environment: 1

Overall Impact: This is an excellent proposal that seeks to evaluate the safety and feasibility of administering IVMg to ED pediatric patients with severe acute asthma. The proposal addresses a significant problem and contains several elements of innovation. The team of investigators is excellent and the environment is exceptional, leveraging existing PECARN infrastructure. Study design and statistical analysis plans are adequate, well developed and well harmonized.

1. Significance:

Strengths

- Finding reliable ways to reduce hospitalization rates for ED pediatric patients with severe acute asthma is very important.
- The planned subsequent clinical trial mirrors the structure of the proposed pilot trial and will be powered to detect significant differences among the three arms.

Weaknesses

- None noted.

2. Investigator(s):

Strengths

- The team of investigators is highly-qualified to conduct this research.
- Statistical support for the pilot and the planned future trial is very solid.

Weaknesses

- None noted.

3. Innovation:

Strengths

- The innovative aspects of this proposal consist of the improved blood sample collection with less pain, the design aimed at identifying an optimal IVMg dose guided via PK/PD modeling and the planned early administration of IVMg in ED.
- The use of phase adaptation as a key design aspect in the future trial is innovative.

Weaknesses

- None noted.

4. Approach:

Strengths

- The statistical analysis plan is extremely well-developed.
- Adequate attention is paid to important analytical aspects, such as the handling of missing data.
- Power calculations are adequate and well-aligned with the study objective and design.
- Leveraging the already existing PECARN infrastructure will further facilitate the conduct of the pilot and planned future trial.
- The use of a common design structure for the pilot and planned future trial will permit the investigators to work out some of the key practical aspects of this research.

Weaknesses

- None noted.

Study Design:

Strengths

- The study design is well-justified and well-reasoned and will provide valuable insight.

- The use of phase adaptation in the planned future trial will benefit efficiency as it will require fewer participants.
- Plans for enrollment and retention are adequate.

Weaknesses

- None noted.

Data Management and Statistical Analysis:

Strengths

- Data management plans are excellent and leverage the PECARN DCC infrastructure.
- The ways in which pilot trial conduct will inform the scale and conduct of the planned future trial are clearly spelled out and thought-out.

Weaknesses

- None noted.

5. Environment:

Strengths

- The environment is exceptional, and leveraging the PECARN infrastructure will not only facilitate enrollment, but also the overall conduct of this research.

Weaknesses

- None noted.

Study Timeline:

Strengths

- Study timelines are detailed and appropriate.
- The use of PECARN resources will increase efficiency on multiple fronts, including enrollment, study conduct, data transmission, storage and analysis

Weaknesses

- None noted.

Protections for Human Subjects:

Acceptable Risks and/or Adequate Protections

Data and Safety Monitoring Plan (Applicable for Clinical Trials Only):

Acceptable

- A five-person DSMB will be assembled.

Inclusion Plans:

- Sex/Gender: Distribution justified scientifically
- Race/Ethnicity: Distribution justified scientifically
- For NIH-Defined Phase III trials, Plans for valid design and analysis: Scientifically acceptable

- Inclusion/Exclusion Based on Age: Distribution justified scientifically

Resource Sharing Plans:

Acceptable

Authentication of Key Biological and/or Chemical Resources:

Not Applicable (No Relevant Resources)

Budget and Period of Support:

Recommend as Requested

(End of Reviewers' Comments)

THE FOLLOWING SECTIONS WERE PREPARED BY THE SCIENTIFIC REVIEW OFFICER TO SUMMARIZE THE OUTCOME OF DISCUSSIONS OF THE REVIEW COMMITTEE, OR REVIEWERS' WRITTEN CRITIQUES, ON THE FOLLOWING ISSUES:

PROTECTION OF HUMAN SUBJECTS: ACCEPTABLE
No concerns were raised by the committee.

DATA AND SAFETY MONITORING PLAN (APPLICABLE FOR CLINICAL TRIALS ONLY): ACCEPTABLE
No concerns were raised by the committee.

INCLUSION OF WOMEN PLAN: ACCEPTABLE
No concerns were raised by the committee.

INCLUSION OF MINORITIES PLAN: ACCEPTABLE
No concerns were raised by the committee.

INCLUSION ACROSS THE LIFESPAN PLAN: ACCEPTABLE
No concerns were raised by the committee.

AUTHENTICATION OF KEY BIOLOGICAL AND/OR CHEMICAL RESOURCES: NOT APPLICABLE

RESOURCE SHARING: ACCEPTABLE

COMMITTEE BUDGET RECOMMENDATIONS: The budget was recommended as requested.

Footnotes for 1 R34 HL152047-01A1; PI Name: Johnson, Michael David

NIH has modified its policy regarding the receipt of resubmissions (amended applications).See Guide Notice NOT-OD-18-197 at https://grants.nih.gov/grants/guide/notice-files/NOT-OD-18-197.html. The impact/priority score is calculated after discussion of an application by averaging the overall scores (1-9) given by all voting reviewers on the committee and multiplying by 10. The criterion scores are submitted prior to the meeting by the individual reviewers assigned to an application, and are not discussed specifically at the review meeting

or calculated into the overall impact score. Some applications also receive a percentile ranking. For details on the review process, see http://grants.nih.gov/grants/peer_review_process.htm#scoring.

MEETING ROSTER

NHLBI Single-Site and Pilot Clinical Trials Study Section
Heart, Lung, and Blood Initial Review Group
NATIONAL HEART, LUNG, AND BLOOD INSTITUTE
SSPT (OA)
06/23/2021 - 06/24/2021

Notice of NIH Policy to All Applicants: Meeting rosters are provided for information purposes only. Applicant investigators and institutional officials must not communicate directly with study section members about an application before or after the review. Failure to observe this policy will create a serious breach of integrity in the peer review process, and may lead to actions outlined in NOT-OD-14-073 at https://grants.nih.gov/grants/guide/notice-files/NOT-OD-14-073.html, NOT-OD-15-106 at https://grants.nih.gov/grants/guide/notice-files/NOT-OD-15-106.html, and NOT-OD-18-115 at https://grants.nih.gov/grants/guide/notice-files/NOT-OD-18-115.html, including removal of the application from immediate review.

CHAIRPERSON(S)

SHEA, STEVEN J, MD
HAMILTON SOUTHWORTH PROFESSOR OF MEDICINE
PROFESSOR OF EPIDEMIOLOGY
VAGELOS COLLEGE OF PHYSICIANS AND SURGEONS
COLUMBIA UNIVERSITY
NEW YORK, NY 10032

MEMBERS

AHN, CHUL W, PHD
PROFESSOR AND DIRECTOR
POPULATION AND DATA SCIENCES
BIOSTATISTICS, RESEARCH DESIGNS & SHARED RESOURCES
UNIVERSITY OF TEXAS SOUTHWESTERN MEDICAL CENTER
DALLAS, TX 75390

ANDREI, ADIN-CRISTIAN, PHD *
PROFESSOR
DEPARTMENT OF PREVENTIVE MEDICINE
NORTHWESTERN UNIVERSITY
CHICAGO, IL 60611

BEAVERS, DANIEL P, PHD *
ASSOCIATE PROFESSOR
DEPARTMENT OF BIOSTATISTICAL SCIENCES
WAKE FOREST SCHOOL OF MEDICINE
WINSTON-SALEM, NC 27157

BECKER, RICHARD C, MD
PROFESSOR AND ENDOWED CHAIR
DEPARTMENT OF INTERNAL MEDICINE
DIVISION OF CARDIOVASCULAR & HEMATOLOGY MEDICINE
COLLEGE OF MEDICINE
UNIVERSITY OF CINCINNATI
CINCINNATI, OH 45267

BROUSSEAU, DAVID C, MD
PROFESSOR OF PEDIATRICS
CHIEF, SECTION OF PEDIATRIC EMERGENCY MEDICINE
JON E. VICE ENDOWED CHAIR FOR EMERGENCY MEDICINE
MEDICAL COLLEGE OF WISCONSIN
MILWAUKEE, WI 53226

BUSCH, ANDREW M, PHD
ASSOCIATE PROFESSOR
C/O MINNEAPOLIS MEDICAL RESEARCH FOUNDATION
DEPARTMENT OF MEDICINE
HENNEPIN HEALTHCARE
UNIVERSITY OF MINNESOTA
MINNEAPOLIS, MN 55415

DAS, ABHIK, PHD *
DISTINGUISHED FELLOW IN BIOSTATISTICS
DIVISION OF BIOSTATISTICS AND EPIDEMIOLOGY
RTI INTERNATIONAL
ROCKVILLE, MD 20852

DONAHUE, J. KEVIN, MD
PROFESSOR OF MEDICINE
DEPARTMENT OF MEDICINE
DIVISION OF CARDIOVASCULAR MEDICINE
UNIVERSITY OF MASSACHUSETTS
WORCESTER, MA 01605

FILARDO, GIOVANNI, PHD
ENDOWED CHAIR
DEPARTMENT OF CARDIOVASCULAR EPIDEMIOLOGY
BAYLOR SCOTT & WHITE HEALTH
BAYLOR RESEARCH INSTITUTE
BAYLOR UNIVERSITY MEDICAL CENTER
DALLAS, TX 75214

FREEDLAND, KENNETH E, PHD
PROFESSOR
DEPARTMENT OF PSYCHIATRY AND PSYCHOLOGY
BEHAVIORAL MEDICINE CENTER
WASHINGTON UNIVERSITY
ST. LOUIS, MO 63108

HSUE, PRISCILLA Y, MD *
PROFESSOR
CHIEF OF CARDIOLOGY AT ZSFG
DIVISION OF CARDIOLOGY
DEPARTMENT OF MEDICINE
UNIVERSITY OF CALIFORNIA, SAN FRANCISCO
SAN FRANCISCO, CA 94110

JACKSON, DANIEL J, MD
ASSOCIATE PROFESSOR
SECTION OF ALLERGY, IMMUNOLOGY, & RHEUMATOLOGY
DEPARTMENT OF PEDIATRICS
SCHOOL OF MEDICINE AND PUBLIC HEALTH
UNIVERSITY OF WISCONSIN - MADISON
MADISON, WI 53792

KONG, ALBERTA SUM-YU, MD
DIVISION CHIEF
DIVISION OF ADOLESCENT MEDICINE
DEPARTMENT OF PEDIATRICS
THE UNIVERSITY OF NEW MEXICO
ALBUQUERQUE, NM 87131

MACK, WENDY J, PHD
PROFESSOR
DIVISION OF BIOSTATISTICS
DEPARTMENT OF PREVENTIVE MEDICINE
UNIVERSITY OF SOUTHERN CALIFORNIA
LOS ANGELES, CA 90033

MAGALANG, ULYSSES J, MD
PROFESSOR OF INTERNAL MEDICINE
DIVISION OF PULMONARY, CRITICAL CARE
AND SLEEP MEDICINE
COLLEGE OF MEDICINE
THE OHIO STATE UNIVERSITY
COLUMBUS, OH 43210

MARTINEZ, FERNANDO J, MD
PROFESSOR AND DIVISION CHIEF
DIVISION OF PULMONARY AND CRITICAL CARE MEDICINE
DEPARTMENT OF MEDICINE
WEILL CORNELL MEDICAL COLLEGE
CORNELL UNIVERSITY
NEW YORK, NY 10065

MOISE, NATHALIE, MD *
FLORENCE IRVING ASSISTANT PROFESSOR OF MEDICINE
DIVISION OF GENERAL MEDICINE
DIRECTOR OF IMPLEMENTATION SCIENCE
CENTER FOR BEHAVIORAL CARDIOVASCULAR HEALTH
COLUMBIA UNIVERSITY IRVING MEDICAL CENTER
NEW YORK, NY 10025

PENG, LIMIN, PHD
PROFESSOR
DEPARTMENT OF BIOSTATISTICS AND BIOINFORMATICS
ROLLINS SCHOOL OF PUBLIC HEALTH
EMORY UNIVERSITY
ATLANTA, GA 30322

PIANTADOSI, STEVEN, MD, PHD
PROFESSOR
DEPARTMENT OF SURGERY
BRIGHAM AND WOMEN'S HOSPITAL
BOSTON, MA 02115

QUYYUMI, ARSHED A, MD
PROFESSOR OF MEDICINE
DIVISION OF CARDIOLOGY
CLINICAL CARDIOVASCULAR RESEARCH INSTITUTE
EMORY UNIVERSITY SCHOOL OF MEDICINE
ATLANTA, GA 30322

SESSO, HOWARD D, MPH, SCD
ASSOCIATE PROFESSOR
DIVISION OF PREVENTIVE MEDICINE
HARVARD MEDICAL SCHOOL
BRIGHAM AND WOMEN'S HOSPITAL
BOSTON, MA 02115

SHARKOSKI, TIFFANY, MPH, MBE *
ASSOCIATE DIRECTOR OF RESEARCH
DIVISION OF CARDIOLOGY
PERELMAN SCHOOL OF MEDICINE
UNIVERSITY OF PENNSYLVANIA
PHILADELPHIA, PA 19104-6021

STEFAN, MIHAELA S, MD, PHD
ASSOCIATE PROFESSOR
DEPARTMENT OF MEDICINE
BAYSTATE MEDICAL CENTER
UNIVERSITY OF MASSACHUSETTS
SPRINGFIELD, MA 01199

STORROW, ALAN B, MD
PROFESSOR
DEPARTMENT OF EMERGENCY MEDICINE
RESEARCH AND ACADEMIC AFFAIRS
VANDERBILT UNIVERSITY MEDICAL CENTER
NASHVILLE, TN 37232

SULLIVAN, DEBRA K, PHD
PROFESSOR AND CHAIR
DEPARTMENT OF DIETETICS AND NUTRITION
MIDWEST DAIRY COUNCIL IN CLINICAL NUTRITION
UNIVERSITY OF KANSAS MEDICAL CENTER
KANSAS CITY, KS 66160

SCIENTIFIC REVIEW OFFICER

LI-SMERIN, YINGYING, MD, PHD
SCIENTIFIC REVIEW OFFICER
OFFICE OF SCIENTIFIC REVIEW/DERA
NATIONAL HEART, LUNG, AND BLOOD INSTITUTE
BETHESDA, MD 20892-7924

EXTRAMURAL SUPPORT ASSISTANT

FELDER, ROBIN
STAFF ASSISTANT
OFFICE OF SCIENTIFIC REVIEW/DERA
NATIONAL HEART, LUNG, AND BLOOD INSTITUTE
NATIONAL INSTITUTES OF HEALTH
BETHESDA, MD 20892

PROGRAM REPRESENTATIVE

BAIZER, LAWRENCE, PHD
SCIENTIFIC REVIEW OFFICER
CENTER FOR SCIENTIFIC REVIEW
NATIONAL INSTITUTES OF HEALTH
BETHESDA, MD 20892

BOYINGTON, JOSEPHINE, PHD
HEALTH SCIENTIST ADMINISTRATOR
DIVISION OF CARDIOVASCULAR SCIENCES
NATIONAL HEART, LUNG, AND BLOOD INSTITUTE
NATIONAL INSTITUTE OF HEALTH
BETHESDA, MD 20892-4870

BROWN, ALISON, PHD
DIVISION OF CARDIOVASCULAR SCIENCES
NATIONAL HEART, LUNG, AND BLOOD INSTITUTE
NATIONAL INSTITUTES OF HEALTH
BETHESDA, MD 20892

CAMPO, REBECCA A, PHD
PROGRAM DIRECTOR
DIVISION OF CARDIOVASCULAR SCIENCES
NATIONAL HEART, LUNG AND BLOOD INSTITUTE
NATIONAL INSTITUTES OF HEALTH
BETHESDA, MD 20892

CRAIG, MATT, PHD
PROGRAM OFFICER
DIVISION OF LUNG DISEASES
NATIONAL HEART, LUNG AND BLOOD INSTITUTE
NATIONAL INSTITUTES OF HEALTH
BETHESDA, MD 20892

DANTHI, NARASIMHAN, PHD
PROGRAM DIRECTOR
ADV.TECH. AND SURGERY BRANCH
DIVISION OF CARDIOVASCULAR SCIENCES
NATIONAL HEART, LUNG, AND BLOOD INSTITUTE
NATIONAL INSTITUTES OF HEALTH
BETHESDA, MD 20892

EGERSON, D'ANDREA R
CLINICAL TRIALS SPECIALIST
HEART DEVELOPMENT AND STRUCTURAL DISEASES BRANCH
NATIONAL HEART LUNG AND BLOOD INSTITUTE
NATIONAL INSTITUTES OF HEALTH
BETHESDA, MD 20817

EL KASSAR, NAHED, MD, PHD
MEDICAL OFFICER
DIVISION OF BLOOD DISEASES AND RESOURCES
NATIONAL HEART, LUNG, AND BLOOD INSTITUTE
NATIONAL INSTITUTES OF HEALTH
BETHESDA, MD 20892

FINE, LARRY, MD
BRANCH CHIEF
DIVISION OF CARDIOVASCULAR SCIENCES
CLINICAL APPLICATIONS AND PREVENTION BRANCH
NATIONAL HEART, LUNG AND BLOOD INSTITUTE
NATIONAL INSTITUTES OF HEALTH
BETHESDA, MD 20892

FREEMER, MICHELLE M, MD
PROGRAM REPRESENTATIVE
DIVISION OF LUNG DISEASES
NATIONAL HEART, LUNG AND BLOOD INSTITUTE
NATIONAL INSTITUTES OF HEALTH
BETHESDA, MD 20892

NATARAJAN, ARUNA R, MD, PHD
MEDICAL OFFICER
LUNG BIOLOGY AND DISEASE PROGRAM
NATIONAL HEART, LUNG AND BLOOD INSTITUTE
NATIONAL INSTITUTES OF HEALTH
BETHESDA, MD 20892-7952

POSTOW, LISA, PHD
PROGRAM OFFICER
AIRWAY BIOLOGY AND DISEASE PROGRAM
NATIOANAL HEART, LUNG, AND BLOOD INSTITUTE
NATIONAL INSTITUTES OF HEALTH
BETHESDA, MD 20892

PRATT, CHARLOTTE, PHD
HEALTH SCIENTIST ADMINISTRATOR
PROGRAM DIRECTOR
DIVISION OF EPIDEMIOLOGY & CLINICAL APPLICATIONS
NATIONAL HEART, LUNG AND BLOOD INSTITUTE
NATIONAL INSTITUTES OF HEALTH
BETHESDA, MD 20892

REDMOND, NICOLE MD, MD
MEDICAL OFFICER
CLINICAL APPLICATIONS AND PREVENTION BRANCH
NATIONAL HEART, LUNG AND BLOOD INSTITUTE
NATIONAL INSTITUTES OF HEALTH
BETHESDA, MD 20817

REINECK, LORA A, MD
MEDICAL OFFICER
DIVISION OF LUNG DISEASES
NATIONAL HEART, LUNG, AND BLOOD INSTITUTE
NATIONAL INSTITUTES OF HEALTH
BETHESDA, MD 20892

ROPER, REBECCA, MPH
SCIENTIFIC REVIEW OFFICER
CENTER FOR TRANSLATION RESEARCH AND IMPLEMENTATION SCIENCE
NATIONAL HEART, LUNG, AND BLOOD INSTITUTE
NATIONAL INSTITUTES OF HEALTH
ROCKVILLE, MD 20857

SCHOPFER, DAVID WESLEY
PHYSICAN
ATHEROTHROMBOSIS & CORONARY ARTERY DISEASE BRANCH
NATIONAL HEART, LUNG, AND BLOOD INSTITUTE
NATIONAL INSTITUTES OF HEALTH
BETHESDA, MD 20892

SOPKO, GEORGE, MD, MPH
MEDICAL OFFICER
DIVISION OF HEART AND VASCULAR DISEASES
NATIONAL HEART, LUNG, AND BLOOD INSTITUTE
NATIONAL INSTITUTES OF HEALTH
BETHESDA, MD 20892

TINSLEY, EMILY
PROGRAM REPRESENTATIVE
DIVISION OF CARDIOVASCULAR DISEASES
NATIONAL HEART, LUNG, AND BLOOD INSTITUTE
NATIONAL INSTITUTES OF HEALTH
BETHESDA, MD 20892

VUGA, LOUIS J, MD, PHD
PROGRAM OFFICER
DIVISION OF LUNG DISEASES
NATIONAL HEART, LUNG, AND BLOOD INSTITUTE
NATIONAL INSTITUTES OF HEALTH
BETHESDA, MD 20892

XIAO, LEI, MD, PHD
MEDICAL OFFICER & PROGRAM DIRECTOR
DIVISION OF LUNG DISEASES
NATIONAL HEART LUNG, AND BLOOD INSTITUTE
NATIONAL INSTITUTES OF HEALTH
BETHESDA, MD 20892

ZOU, SHIMIAN, PHD
PROGRAM OFFICER
BLOOD EPIDEMIOLOGY & CLINICAL THERAPEUTICS BRANCH
NATIONAL HEART, LUNG AND BLOOD INSTITUTE
NATIONAL INSTITUTES OF HEALTH
BETHESDA, MD 20892

GRANTS MANAGEMENT REPRESENTATIVE

AGRESTI, TONY
GRANT MANAGEMENT SPECIALIST
OFFICE OF GRANT MANAGEMENT/DERA
NATIONAL HEART, LUNG, AND BLOOD INSTITUTE
NATIONAL INSTITUTES OF HEALTH
BETHESDA, MD 20892

RUNDHAUGEN, LYNN M
GRANTS MANAGEMENT SPECIALIST
OFFICE OF GRANTS MANAGEMENT/DERA
NATIONAL HEART, LUNG, AND BLOOD INSTITUTE
NATIONAL INSTITUTES OF HEALTH
BETHESDA, MD 20892-7956

SIMPSON, TAMMI
GRANT MANAGEMENT SPECIALIST
OFFICE OF GRANTS MANAGEMENT/DERA
NATIONAL HEART, LUNG, AND BLOOD INSTITUTE
NATIONAL INSTITUTES OF HEALTH
BETHESDA, MD 20892

* Temporary Member. For grant applications, temporary members may participate in the entire meeting or may review only selected applications as needed.

Consultants are required to absent themselves from the room during the review of any application if their presence would constitute or appear to constitute a conflict of interest.
